# Real-time detection method for Litchi diseases and pests based on improved YOLOv5s

**DOI:** 10.3389/fpls.2025.1686997

**Published:** 2025-10-29

**Authors:** Xingzao Ma, Tianyang Huang, Gaoyuan Zhao, Zhi Qiu, Hua Li, Zhuangdong Fang

**Affiliations:** ^1^ School of Mechatronic Engineering, Lingnan Normal University, Zhanjiang, China; ^2^ Shanwei Academy of Agricultural Sciences, Shanwei, China

**Keywords:** Litchi, pests and diseases, real-time detection, YOLOv5S, Raspberry Pi

## Abstract

Accurate, efficient, and economical detection of Litchi pests and diseases is critical for sustainable orchard management, yet traditional manual methods often fall short in these aspects. To address these limitations, an improved YOLOv5s model, named YOLOv5s-SNV2-GSE, was proposed in this study for real-time detection on embedded platforms. The backbone network was modified by replacing conventional convolutional blocks with ShuffleNetV2, leveraging channel shuffling and group convolution to reduce model parameters and computational cost. In the detection head, standard convolutional blocks and C3 modules were replaced with depthwise convolutions (DWConv) and C3Ghost modules to further minimize model size. Squeeze-and-Excitation (SE), Convolutional Block Attention Module (CBAM), and Coordinate Attention (CoordAtt) mechanisms were incorporated into the backbone network to enhance feature extraction. Additionally, the Efficient Intersection over Union (EIoU) loss function was adopted to improve convergence speed and bounding box regression accuracy. The experimental results demonstrated that the improved YOLOv5s-SNV2-GSE model achieved a mean average precision (mAP) of 96.7%. Compared to the original YOLOv5s, the proposed model reduced computational cost by 87.5%, number of parameters by 86.7%, and model size by 55.6%. When deployed on a Raspberry Pi 4B, the model achieved an average inference speed of 3.3 frames per second (FPS), representing a 57.1% improvement and meeting real-time detection requirements. These results indicate that the proposed model provides a practical and efficient solution for real-time Litchi pests and diseases detection in resource-constrained environments.

## 1 Introduction

China is the world leader in both the planting area and yield of Litchi, particularly in Guangdong Province, where Litchi cultivation is not only a local specialty industry but also plays an important role in the global market (Zhuang and Qiu, 2021; Xiang, 2020). However, Litchi is highly vulnerable to pests and diseases stress during growth, which can significantly reduce yield and fruit quality, and in severe cases, lead to plant death (Huang, 2021). Therefore, establishing an efficient and precise pests and diseases monitoring and control system has become an urgent need for the sustainable development of the Litchi industry. Traditional manual inspection and visual diagnosis methods are limited by low efficiency and strong subjectivity, which makes deep learning-based intelligent detection technologies a research hotspot in the field of agricultural engineering.

The rapid development of deep learning (DL) techniques, particularly Convolutional Neural Networks (CNNs), has provided new technological support for the intelligent detection of crop pests and diseases (Xu et al., 2023; Guo et al., 2025). In recent years, significant progress has been made in deep learning (DL)-based pests and diseases monitoring and diagnosis, with extensive research conducted by both domestic and international scholars (Zhou et al., 2024; Li et al., 2024; Shoaib et al., 2025; Rahman et al., 2020). Tan et al. (2024) developed a mobile real-time citrus disease diagnosis system by integrating the Inceptionv3 backbone network with the CBAM, achieving an accuracy of 98.49%. Ai et al. (2020) designed a crop pests and diseases recognition app based on the Inception-ResNet-v2 model, with an overall recognition accuracy of 86.1%. Waheed et al. (2022) used various DL models to detect ginger pests and diseases, finding that the VGG-16 algorithm achieved an accuracy of 96%. Jelali (2024) utilized CNN, Faster R-CNN, and YOLOv5s models for tomato pest detection, with the CNN model achieving 90% accuracy. Yue et al. (2024) introduced the SCYLLA-IOU loss function in the YOLOv8s model, improving pest detection accuracy to 97.4%, providing a reference for optimizing small target detection models. Xie et al. (2023) adopted G-GhostNet as the backbone network, introduced the Centered Moment Pooling Attention (CMPA) mechanism, and an improved loss function, and constructed the FCOS-FL model, effectively achieving accurate detection of Litchi leaf pests and diseases with an accuracy rate of 91.3%. Despite significant advancements in deep learning for crop pests and diseases detection, research specifically targeting Litchi remains relatively limited. Existing studies have primarily focused on static image-based recognition, with insufficient attention to real-time detection capabilities and deployment on resource-constrained embedded platforms. Traditional models such as Faster R-CNN and VGG-16, for instance, generally incur substantial computational overhead when processing high-dimensional data, rendering them less adaptable to scenarios with stringent real-time requirements (Ren et al., 2017; Simonyan and Zisserman, 2014). In contrast, YOLOv5s distinguishes itself through its highly lightweight architecture. It not only satisfies the deployment demands of embedded platforms but also maintains high detection accuracy. This unique balance of efficiency and precision gives it a distinct competitive edge when compared to other deep learning models such as YOLOv8s and Faster R-CNN. Consequently, improving upon the YOLOv5s framework represents a feasible strategy for this study.

To address the challenges of limited accuracy, low efficiency, and high deployment costs in Litchi pests and diseases detection, this paper proposed an improved YOLOv5s model by integrating a lightweight backbone network and attention mechanisms, thereby developing an efficient and accurate detection model for deployment on the Raspberry Pi 4B embedded platform. The research result not only provides technical support for the green pest control of Litchi but also offers theoretical and practical references for the construction of intelligent monitoring systems for other crop pests and diseases.

## 2 Materials and methods

### 2.1 Litchi pests and diseases dataset acquisition

**Figure 1:**
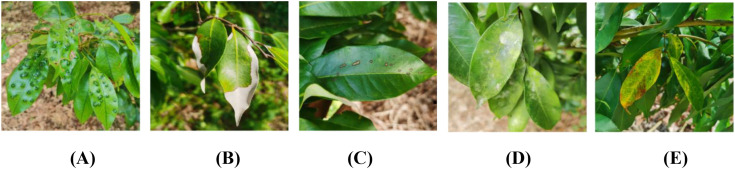
Litchi pests and diseases. **(A)** Dasineura **(B)** Anthracnose **(C)** Algal spot **(D)** Sooty mold **(E)** Ulcer disease.

The dataset used in this study was collected in November 2024 from a Litchi orchard in Lianjiang City, Zhanjiang, Guangdong Province. The temperature ranged from 22°C to 28°C, the weather was clear, and the orchard received abundant natural sunlight. Data collection took place during the daytime, between 9:00 and 15:00. Each image depicts the natural condition of Litchi leaves, consistent with real-world growing environments. The dataset includes five categories of pests and diseases instances, Dasineura, Anthracnose, Algal spot, Sooty mold, and Ulcer disease. These primarily consist of pest infestations, fungal diseases, and algal diseases. The Dasineura involves the female adult laying eggs on the underside or tips of young leaves. Upon hatching, the larvae feed on the leaves, stimulating abnormal cell proliferation, resulting in galls. Fungal diseases such as Anthracnose, Ulcer disease, and Sooty mold require specific temperature and humidity conditions, as well as transmission vectors. Anthracnose mainly affects the leaves, flower clusters, and fruit, causing brown lesions and rot. Sooty mold is associated with pests such as scale insects and aphids, whose secreted sugars provide a breeding substrate for fungi, forming a black mold that impacts photosynthesis. Ulcer disease primarily targets the branches and main trunk. The pathogens enter through wounds, causing necrosis and cracking of the bark. The lesions turn from reddish-brown to gray-brown with a central depression. Algal spot disease, caused by parasitic algae, manifests on the leaf surface as gray-green to yellow-brown spots, with spores being spread by rainwater. To ensure the independence of the dataset, images underwent preprocessing through selection and cropping. Images with artifacts or occlusions by non-target objects were excluded, while those with clear pests and diseases symptoms were retained. Large background areas were cropped to focus on the affected leaf regions, resulting in 545 images used to create the Litchi leaf pests and diseases dataset. Samples of the five pests and diseases are shown in **Figure 1**.

### 2.2 Dataset construction

**Figure 2:**
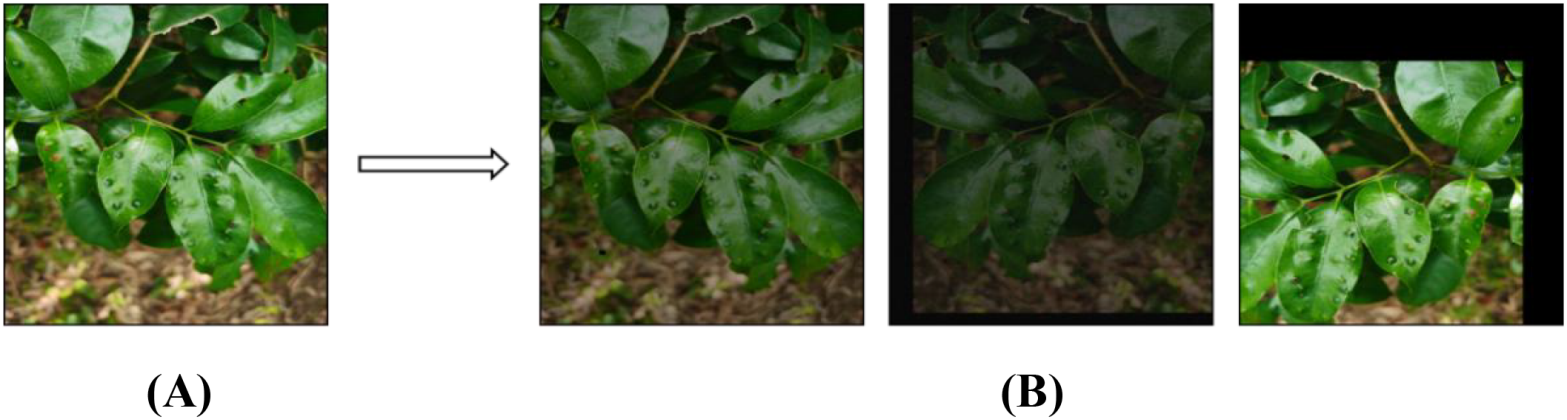
Image enhancement. **(A)** Original image **(B)** Corresponding augmented version.

To improve the model’s generalization ability and robustness, data augmentation was performed on the original dataset. Techniques including mirroring, rotation, brightness adjustment, and noise addition were applied, with at least two methods randomly combined for each sample to enhance the size and diversity of the training set. As a result, the dataset was expanded from 545 to 2,180 images. The distribution of samples across disease categories is presented in **Table 1**. The visual effects of the augmentation are illustrated in **Figure 2**, where **Figure 2A** shows an original image and **Figure 2B** shows the corresponding augmented version.

**Table 1:** Litchi diseases and pests dataset.

Pests and diseases categories	Dasineura	Anthracnose	Algal spot	Sooty mold	Ulcer disease
Original image/sheet	117	112	105	110	101
Enhanced image/sheet	351	336	315	330	303
Total/sheet	468	448	420	440	404

**Figure 3:**
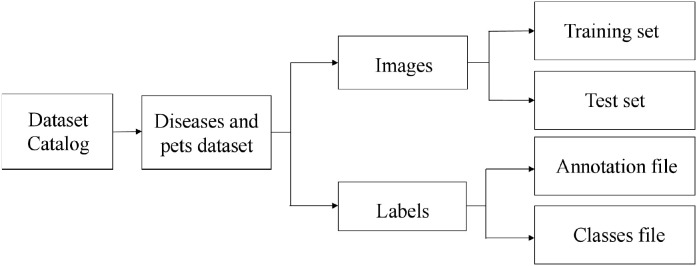
Folder hierarchy diagram.

Each image was annotated using the LabelImage tool (Version 1.2.2), with labels for Dasineura, Anthracnose, Algal spot, Sooty mold, and Ulcer disease being “yeyingwen”, “tanjv”, “zaoban”, “meiyan” and “kuiyang”, respectively. The generated target information was stored in the corresponding TXT files. The dataset was split into training, validation, and test sets in a 7:2:1 ratio, as shown in **Table 2**. The dataset structure was illustrated in the folder hierarchy in **Figure 3**, with the “images” and “labels” folders included in the dataset directory. The “images” folder contains the images required for training and testing, while the “labels” folder contains the annotation files and class names.

**Table 2:** Division of Litchi pests and diseases dataset.

Pests and diseases categories	Training set/sheet	Verification set/sheet	Test set/sheet	Total/sheet
Dasineura	328	93	47	468
Anthracnose	314	89	45	448
Algal spot	294	84	42	420
Sooty mold	308	88	44	440
Ulcer disease	283	81	40	404

### 2.3 Models and training

#### 2.3.1 Improved YOLOv5s model

The YOLO (You Only Look Once) algorithm, proposed by Joseph Redmon and others in 2016, abandons the traditional multi-stage process with its innovative regression approach, directly predicting the class and spatial location of objects in images in an end-to-end manner, making it a highly popular and efficient algorithm in real-time object detection (Alhwaiti et al., 2025; Zhu et al., 2023; Liu et al., 2023). YOLOv5s, as an important evolutionary version of this series, has undergone various optimizations in its model architecture and training strategies (Wang et al., 2022; Redmon et al., 2016; Redmon and Farhadi, 2018). Compared to previous versions, YOLOv5s adopts a more lightweight network structure, fully considering the constraints of computational resources. By optimizing computational load and storage overhead, it reduces hardware requirements while maintaining high-performance object detection. This makes YOLOv5s suitable for deployment on embedded and mobile devices, especially in scenarios with limited computational resources. YOLOv5s also incorporates techniques such as adaptive learning rate adjustment and multi-scale training to further improve the model’s training effectiveness. These innovative data augmentation strategies and training techniques enable YOLOv5s to achieve high accuracy and robustness in real-time object detection tasks.

**Figure 4:**
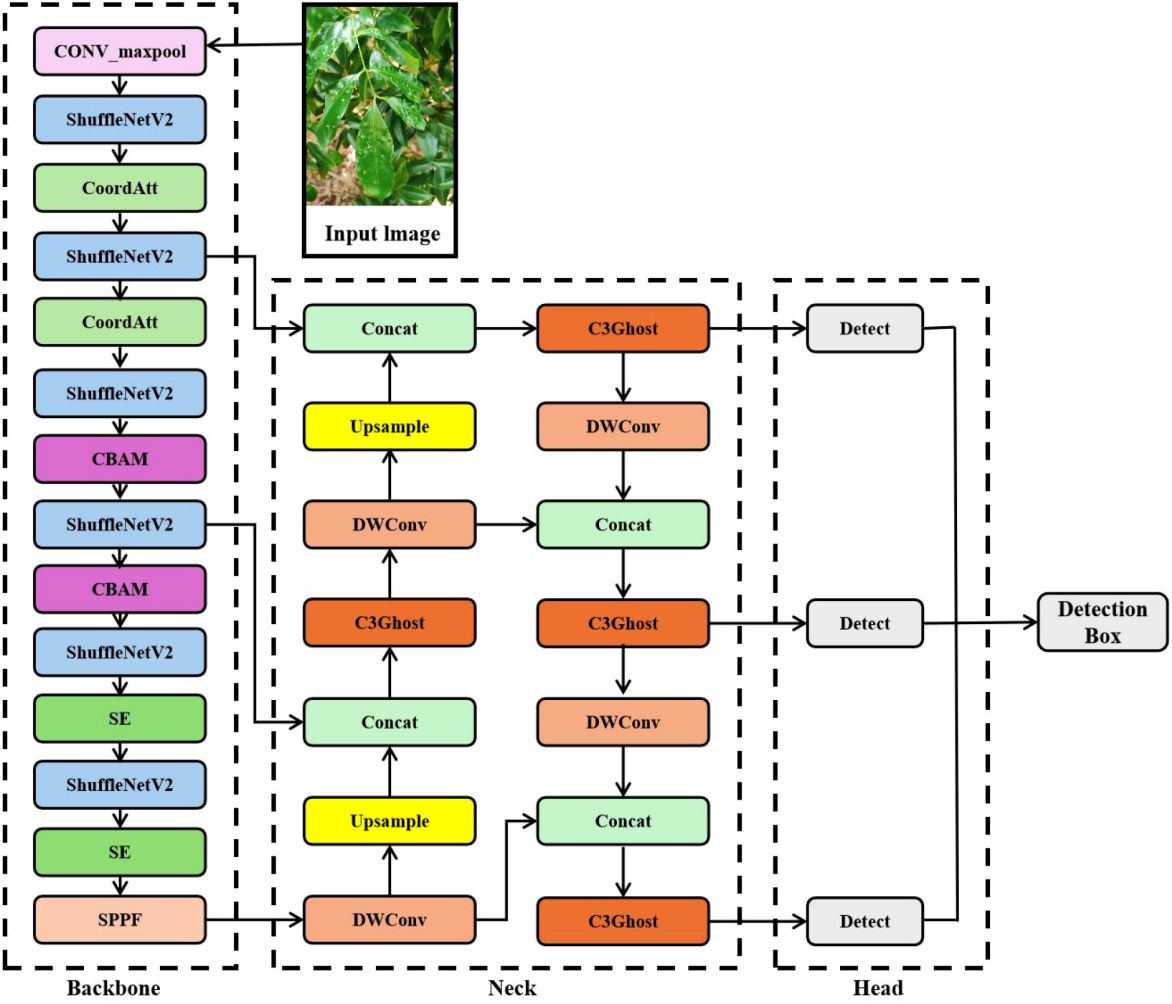
Model network structure diagram.

Although the YOLOv5s model demonstrates strong overall performance in feature extraction and model size, it still faces the challenge of insufficient computing power when deployed on embedded devices. To address these limitations, this paper proposed an improved version, the YOLOv5s-SNV2-GSE model, to further optimize its computational efficiency while addressing real-time requirements. The network structure is shown in **Figure 4**.

1. To address the issues of feature redundancy and computational resource consumption in the YOLOv5s model, this paper introduced two lightweight architectures, ShuffleNet V2 and MobileNetV3, to replace the original convolution module and optimize the backbone network structure.
2. The detection head network was further optimized by incorporating DWConv and the C3Ghost module for lightweight processing. DWConv reduced parameters and computational load compared to standard convolution, making it commonly used for model simplification. The C3Ghost module combined the structural advantages of the C3 and Ghost modules, enabling further model lightweighting without compromising representational capacity.
3. This paper adopted a hybrid attention integration strategy, selecting appropriate attention mechanisms according to the functional characteristics of different network layers. In the shallow layers, the CoordAtt was employed to enhance spatial feature representation. In the intermediate layers, the CBAM was applied to jointly refine spatial and channel-wise features. In the deep layers, the SE mechanism was utilized to strengthen channel-wise feature recalibration. This layer-adaptive approach enabled targeted enhancement of feature representation across the network hierarchy.
4. To further optimize the model, this paper employed the EIoU loss function to improve the accuracy of bounding box regression. EIoU optimized the model’s regression accuracy across targets of different scales and shapes, effectively avoiding bounding box misalignment or localization errors that might occur with traditional methods.

#### 2.3.2 YOLOv5s model optimization

##### 2.3.2.1 Introduction of lightweight modules

To address issues such as feature redundancy and excessive parameters in the YOLOv5s model, this paper introduced the lightweight ShuffleNetV2 and MobileNetV3 modules into the YOLOv5s network architecture to reduce the model’s computational intensity and build an efficient object detection model suitable for mobile devices (Bochkovskiy et al., 2020). The ShuffleNet series, proposed by Megvii Technology, aims to solve the problem of limited computational power on mobile devices. Its design philosophy involves reducing computation through channel shuffle and group convolutions while maximizing the model’s computational efficiency. ShuffleNetV2 reduces the computational load of convolution operations, maintaining high expressive power while lowering computational costs. MobileNetV3, a lightweight network architecture proposed by Google, combines the advantages of Neural Architecture Search (NAS) and manual design. By introducing lightweight attention mechanisms and efficient feature extraction modules, it effectively improves performance on mobile devices.

##### 2.3.2.2 Head network optimization

To further reduce the computational burden of the YOLOv5s model, this paper optimized the detection head by incorporating two lightweight modules—DWConv and the C3Ghost module—to replace standard convolutional operations. DWConv, a widely adopted operator in efficient neural networks, decouples spatial and channel-wise feature extraction: it first applies depthwise convolutions to filter each input channel independently, followed by 1×1 pointwise convolutions to fuse the outputs across channels. This factorized approach significantly reduced both the number of parameters and computational cost (Zhang et al., 2017).

**Figure 5:**
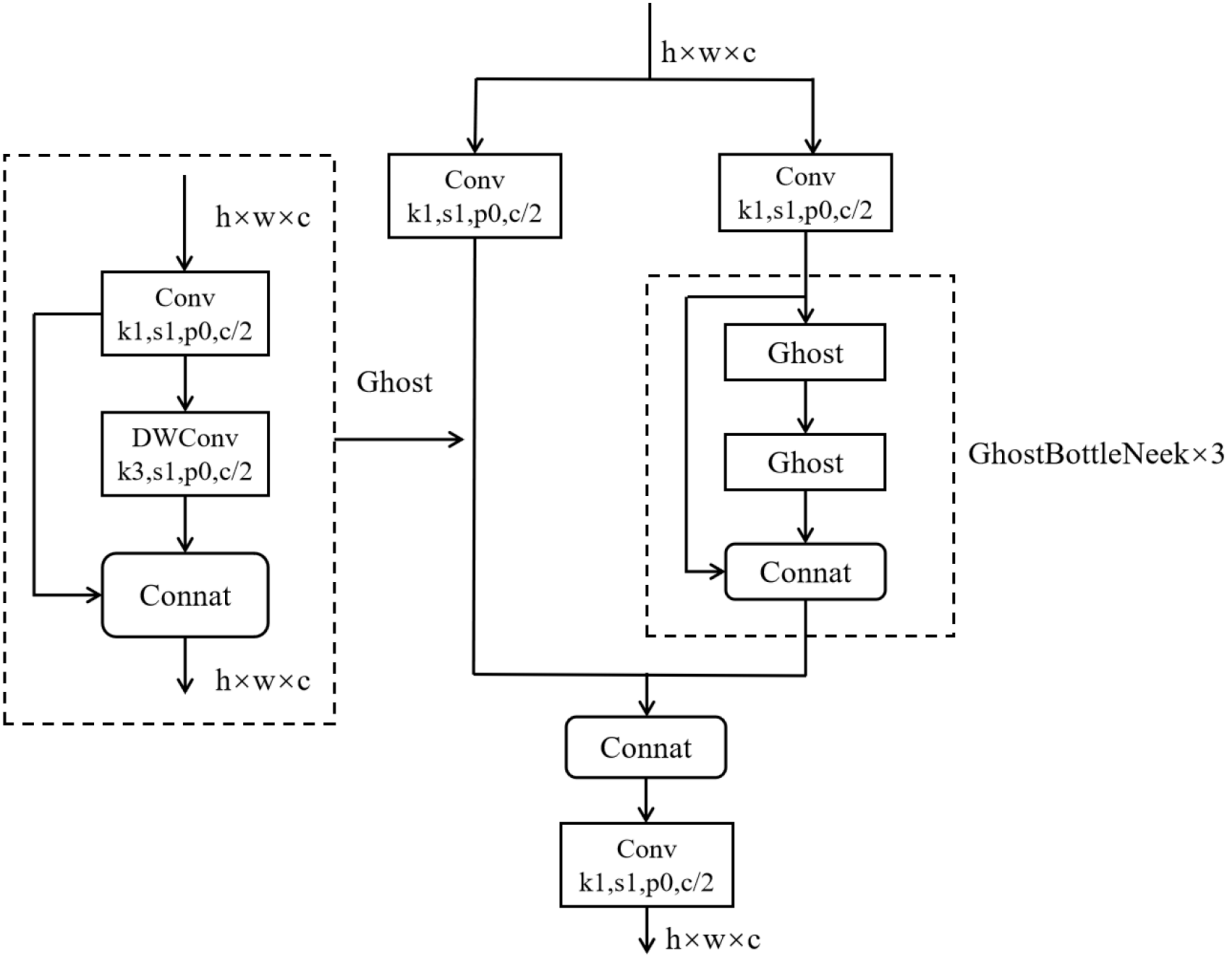
C3Ghost structural diagram.

The C3Ghost module is constructed based on the Ghost module, which consists of traditional convolution and DWConv, combining the advantages of both the C3 module and the Ghost module. As shown in **Figure 5**, the C3 module extracts features through convolution layers with residual connections, offering strong feature representation capabilities. The Ghost module, on the other hand, generates redundant feature maps through low-cost operations, effectively reducing the computational burden. Therefore, by incorporating the Ghost module into the C3 module, the C3Ghost module retains the original feature extraction capabilities while further reducing computation and parameter counts, thereby enhancing the model’s lightweight effect.

##### 2.3.2.3 Attention mechanism

The attention module enables convolutional neural networks to focus more on the key information in the data, reducing the interference from irrelevant content and noise. By dynamically calculating channel and spatial weights, it enhances the model’s sensitivity to subtle features and small targets, thereby improving detection accuracy and robustness, especially in complex scenarios, effectively reducing false negatives (FN) and false positives (FP). This paper introduced three attention mechanisms SE, CoordAtt, and CBAM based on the differences in network depth and location, adapting to the feature processing needs of different layers.

The SE attention mechanism is processed in three stages of feature compression, weight prediction, and response calibration. By modeling the nonlinear dependencies between feature channels, it builds an adaptive weight distribution system. This module dynamically generates the significance weights of each channel through global feature aggregation and gated activation functions, enhancing the important feature channels and suppressing the less important ones (Zhao et al., 2025). Embedding the SE module into the deep network enables the adjustment of global channel weights, which highlights feature channels strongly correlated with pests and diseases categories while suppressing background interference channels. This provides more accurate feature support for subsequent object detection tasks.

CoordAtt adopts a coordinate decomposition strategy by processing the spatial position encoding of the channel dimension in parallel, generating a direction-aware attention weight matrix, and enhancing spatial-sensitive features through feature point multiplication (Hu et al., 2019). The advantage of CoordAtt lies in its ability to significantly reduce computational resources while participating in large-scale modeling for mobile networks. Integrating the CoordAtt module into the shallow layers enables precise localization of fine-grained pests and diseases features through direction-aware spatial weighting. Simultaneously, it suppresses background interference from visually similar textures, providing cleaner local feature representations for subsequent deep feature extraction. Moreover, its inherently low computational overhead avoids increasing the inference burden on the early stages of the network, thereby preserving the model’s real-time inference capability.

**Figure 6:**
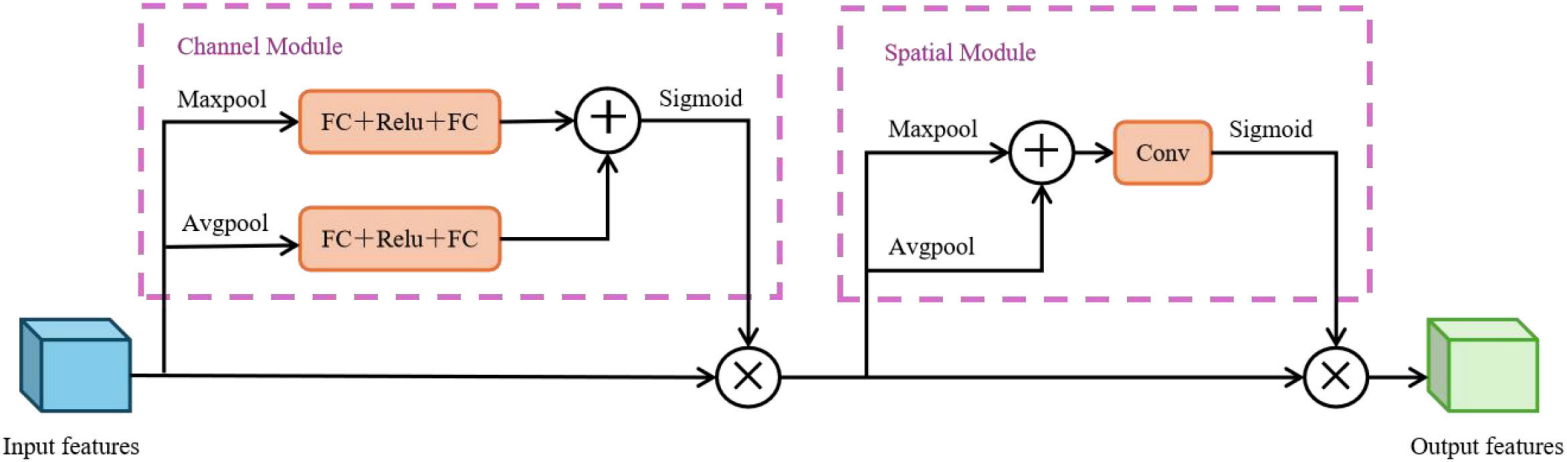
CBAM structural diagram.

The CBAM attention mechanism consists of channel and spatial attention modules. It integrates output features through element-wise multiplication, generating enhanced features with both channel and spatial adaptive filtering capabilities, as shown in **Figure 6**. These features retain significant semantic information, suppress redundant textures and environmental interference, and improve the efficiency of extracting key features. The collaborative optimization of both channel and spatial dimensions strengthens the model’s ability to analyze complex scenarios (Woo et al., 2018). Incorporating the CBAM into the middle network achieves the selection of pest- and disease-related features and accurate localization of their spatial ranges via the collaborative optimization of channel and spatial dimensions. This not only balances the local details from the shallow network but also lays a high-quality feature foundation for subsequent deep feature fusion.

By embedding these three attention modules at different network levels, this paper systematically enhanced the model’s feature perception and utilization capabilities from the perspectives of channel, spatial, and fusion dimensions, providing a better feature processing solution for detection tasks in complex scenarios.

##### 2.3.2.4 Optimization of border loss function

The loss function is a key metric for evaluating the matching degree between the predicted and ground truth bounding boxes. In the field of object detection, most algorithms used the Intersection over Union (IoU) as the standard performance measure for the loss function (Hou et al., 2021). However, IoU only reflects the proportion of the overlapping area between two bounding boxes. When the ground truth and predicted boxes are partially or completely non-overlapping, IoU fails to effectively quantify the spatial consistency error and the normalized center distance between them. This leads to ineffective gradient updates during model training, hindering the convergence process. To address this issue, the YOLOv5s model uses the Complete Intersection over Union (CIoU) as the loss function, significantly improving the model’s localization performance. The CIoU loss function integrates three features: the Euclidean distance between the center points of the target boxes, aspect ratio consistency, and IoU, forming a multi-dimensional evaluation system for bounding box matching (Yu et al., 2016). However, CIoU does not fully account for the nonlinear relationship between the bounding box geometric dimensions and localization confidence. The EIoU loss function further optimizes this issue by introducing a more detailed multi-dimensional loss evaluation mechanism. It not only considers the overlap of the bounding boxes but also enhances the fine-tuning of the shape and position of the target boxes, resulting in more stable and accurate model performance in complex scenarios (Zhang et al., 2021).

#### 2.3.3 Model training platform and evaluation

##### 2.3.3.1 Model training platform

The training platform used in this paper was configured as follows: the hardware platform included an Intel Core i7 CPU and an NVIDIA GeForce RTX 4090D GPU. The software platform ran on the Ubuntu 20.04 operating system, with CUDA 11.3, Python 3.8, PyTorch 1.10.0, OpenCV 3.4, and Torch 1.6.0 installed.

##### 2.3.3.2 Training parameters

Training parameters were set as follows: the default input image size was 640×640, the batch size was 32, the initial learning rate was set to 0.01, and the training ran for 200 iterations.

##### 2.3.3.3 Evaluation indicators

Deep learning models such as Faster R-CNN and YOLOv5s are commonly evaluated in object detection tasks using core performance metrics including IoU, Precision (P), Recall (R), Average Precision (AP), and mean Average Precision (mAP). These metrics are used to measure the accuracy and completeness of the model’s detection results. In contrast, the evaluation of model lightweighting is generally reflected by indicators such as Floating Point Operations (FLOPs), parameter count, and model storage size, which reflect the model’s efficiency and resource consumption in practical deployment. Based on this, this paper adopted mAP, FLOPs, parameter count, and model storage size as the performance evaluation metrics, with the calculation of mAP detailed in Equation 1.


(1) $$\left\{ \begin{array}{c} \text{P}=\frac{TP}{TP+FP}\times 100\%\\ \text{R}=\frac{TP}{TP+FN}\times 100\%\\ AP_{k}=\frac{1}{M}\sum_{L=1}^{M}P_{k}(L)\text{Δ}R(L)\\ mAP=\frac{1}{k}\sum_{k=1}^{5}AP_{k} \end{array}\right.$$

where, *TP* refers to the number of correctly identified samples that belong to positive classes and match their actual categories. *FP* is the quantity of false positive samples that are negative classes but are mistakenly judged as positive classes. *FN* is the number of missed samples that are positive classes but are incorrectly classified as negative classes. *k* denotes the number of pests and diseases categories, which is 5. *M* is the number of segments in the recall interval. *P_k_* (*L*) stands for the average precision of the *k*-th category within the *L*-th recall interval. And Δ*R*(*L*) is the length of the *L*-th recall interval.

## 3 Results and analysis

### 3.1 Comparison of lightweight network model performance

The experimental results of the two models obtained after introducing two lightweight architectures are shown in **Table 3**. The YOLOv5s-SNV2 improved model reduced FLOPs, model parameters, and model size by 88.1%, 87.9%, and 58.8%, respectively. The corresponding reductions for the YOLOv5s-MNV3 improved model were 83.7%, 80.2%, and 30.6%. Comparison indicated that the YOLOv5s-SNV2 model had a greater advantage in terms of hardware resource optimization, with a model size reduction 28.2% higher than that of YOLOv5s-MNV3, while the precision loss was only a 2% difference. Although model lightweighting could effectively reduce computational complexity, it might to some extent weaken feature extraction capability, potentially leading to a decrease in model accuracy. Experimental results demonstrated that the ShuffleNetV2-based structural optimization achieves higher efficiency in mobile deployment while preserving detection accuracy within an acceptable margin.

**Table 3:** Comparison test results of YOLOv5s model lightweighting.

Model	mAP/%	FLOPs/G	Parameters/*10^6^	Model size/MB
YOLOv5s	98.5	16	7.03	16
YOLOv5s-SNV2	88.5	1.9	0.85	6.59
YOLOv5s-MNV3	90.5	2.6	1.39	11.1

### 3.2 Model optimization based on YOLOv5s-SNV2

Building upon the lightweight design of the backbone network, this paper further lightweighted the head network by introducing the DWConv and C3Ghost modules. This approach reduced both the computation and parameter count, resulting in the YOLOv5s-SNV2-G model. However, as shown in the experimental results in **Table 3**, although model lightweighting effectively reduced computational complexity, it also led to a significant drop in detection accuracy. To address this issue, we enhanced spatial information in shallow layers and channel information in deep layers according to the network depth. For intermediate layers, a hybrid enhancement strategy was employed to jointly strengthen both spatial and channel representations, leading to the proposed YOLOv5s-SNV2-GS model. In addition, we introduced the EIoU loss function to further optimize the matching between the target boxes and anchor boxes, leading to the YOLOv5s-SNV2-GSE model. The experimental results comparing the three models with YOLOv5s-SNV2 are presented in **Table 4**.

**Table 4:** Performance of the improved model based on YOLOv5s-SNV2.

Model	mAP/%	FLOPs/G	Parameters/*10^6^	Model size/MB
YOLOv5s-SNV2	88.5	1.9	0.85	6.59
YOLOv5s-SNV2-G	85.8	1.4	0.56	4.81
YOLOv5s-SNV2-GS	95.5	2.0	0.93	7.1
YOLOv5s-SNV2-GSE	96.7	2.0	0.93	7.1

As seen in **Table 4**, the YOLOv5s-SNV2-G model showed a decrease in FLOPs, parameter count, model size, and mAP, with reductions of 26.3%, 34.1%, 27%, and 3%, respectively, compared to YOLOv5s-SNV2. However, the YOLOv5s-SNV2-GSE and YOLOv5s-SNV2-GS models showed minimal changes in FLOPs, parameter count, and model size, while their mAP significantly increased to 96.7%. The experimental results demonstrated that the introduction of the hybrid attention mechanism helped the model capture essential features more effectively, improving both processing efficiency and accuracy. At the same time, the EIoU loss function effectively compensated for the discrepancies between the CIoU loss function’s bounding box size and confidence, minimizing the difference between the target box and the anchor box, which significantly enhanced model performance.

### 3.3 Comparison of ablation experiment performance

To evaluate the effectiveness of the proposed improvements and their impact on model performance, this paper conducted ablation experiments, the results presented in **Table 5**. Experiment 1 is the original YOLOv5s model, which achieved an mAP of 98.5% for Litchi pests and diseases detection, with a model size of 16 MB. In Experiment 2, ShuffleNetV2 was used to replace the convolution blocks in the backbone network of YOLOv5s. As a result, mAP decreased to 88.5%, while the model’s FLOPs, parameter count, and size were significantly reduced, with the model size decreasing by 58.8%. In Experiment 3, the standard convolutions in the head network were replaced with DWConv, and the C3 modules were substituted with C3Ghost blocks in the YOLOv5s model. This led to a decrease in mAP to 94.3%, along with a reduction in FLOPs and parameter count. Experiment 4 combined the improvements of ShuffleNetV2, DWConv, and C3Ghost into the YOLOv5s model, resulting in a reduction in FLOPs, parameter count, and model size to 1.4 G, 0.56×10^6^, and 4.81 MB, respectively.

**Table 5:** Results of ablation experiment.

Experiment	ShuffleNetV2	DWCONV、C3Ghost	Fusion attention mechanism	EIoU	mAP/%	FLOPs/G	Parameters/*10^6^	Size/MB
1	–	–	–	–	98.5	16	7.03	16
2	✓	–	–	–	88.5	1.9	0.85	6.59
3	–	✓	–	–	94.3	15.5	6.73	14.3
4	✓	✓	–	–	85.8	1.4	0.56	4.81
5	✓	✓	✓	–	95.5	2.0	0.93	7.1
6	✓	✓	–	✓	86.7	1.4	0.56	4.81
7	✓	✓	✓	✓	96.7	2.0	0.93	7.1

The results of Experiments 1–3 demonstrated that lightweighting improvements could significantly reduce FLOPs, parameter count, and model size, but this often comes at the cost of reduced detection accuracy. In Experiment 5, CBAM, CoordAtt, and SE attention mechanisms were introduced to the lightweight model. Compared with Experiment 4, although the model size and parameters had significantly increased, mAP had been improved, indicating the positive impact of fusion attention mechanism on detection performance. In Experiment 6, the EIoU loss function was integrated into the lightweight model, resulting in a 0.9 percentage point increase in mAP without changing FLOPs, parameter count, or model size, while also accelerating model convergence and improving accuracy. Finally, Experiment 7 integrated all four improvements into the YOLOv5s model. This integrated model achieved a significant reduction in FLOPs and parameter count while preserving high detection accuracy, thereby delivering superior overall performance compared to the original YOLOv5s.

### 3.4 YOLOv5s-SNV2-GSE model performance evaluation

**Figure 7:**
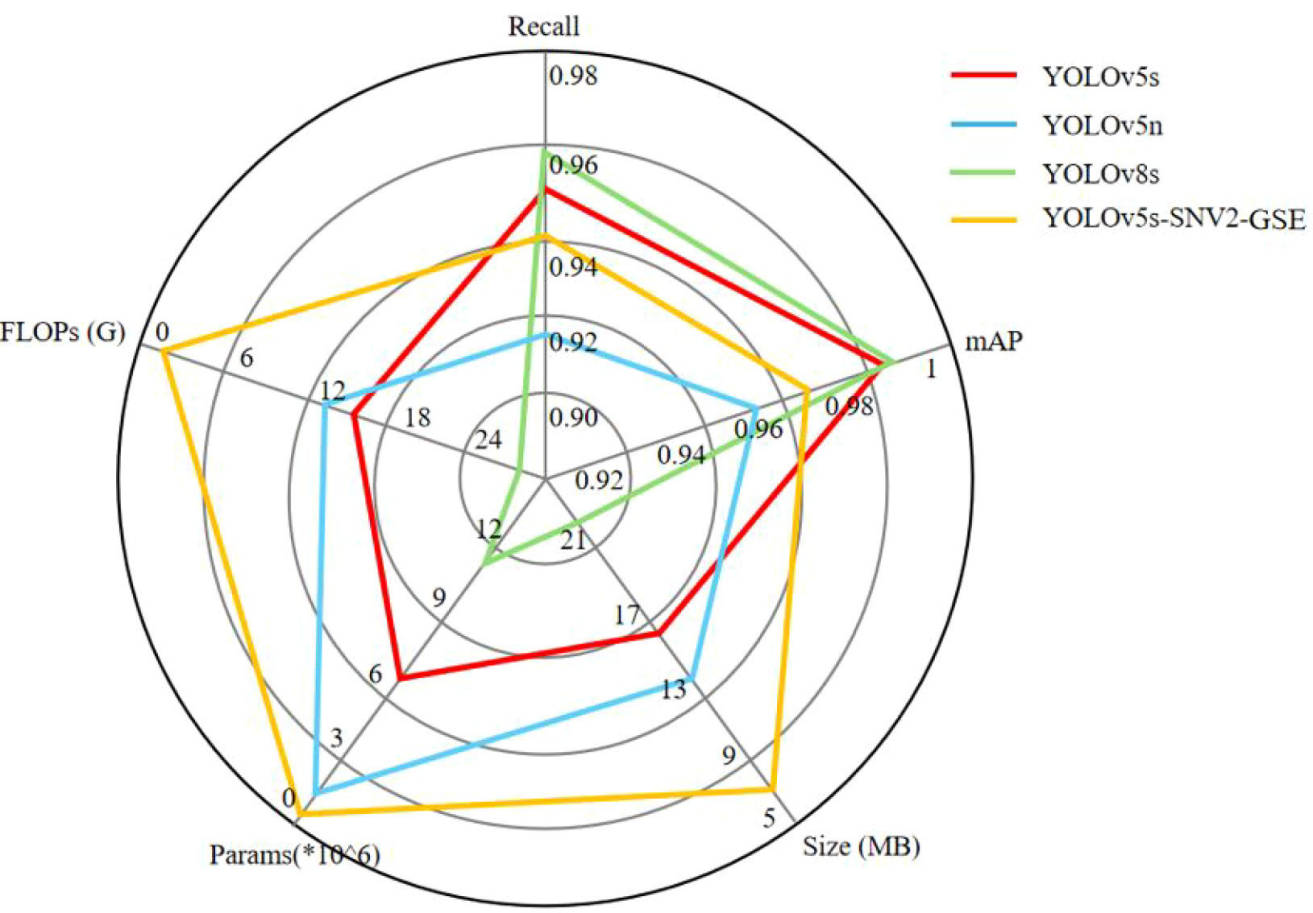
Comparison of model performance.

To evaluate the overall performance of the models, this study conducted comparative experiments on the lightweight algorithms YOLOv5s, YOLOv5n, YOLOv8s, and the proposed YOLOv5s-SNV2-GSE, with the results presented in **Figure 7**. Compared to the original YOLOv5s model, YOLOv5s-SNV2-GSE exhibited only a 1.8% decrease in accuracy, while achieving a 55.6% reduction in model size. Compared with the widely adopted lightweight algorithm YOLOv5n, YOLOv5s-SNV2-GSE demonstrated superior performance in both mAP and model size. While YOLOv8s demonstrated better detection accuracy, its FLOPs and parameter count were significantly higher than those of YOLOv5s-SNV2-GSE, making it unsuitable for mobile deployment. In conclusion, the YOLOv5s-SNV2-GSE model achieved effective lightweight optimization while preserving high detection accuracy, satisfying the computational and storage constraints of mobile device deployments without compromising detection performance.

**Figure 8:**
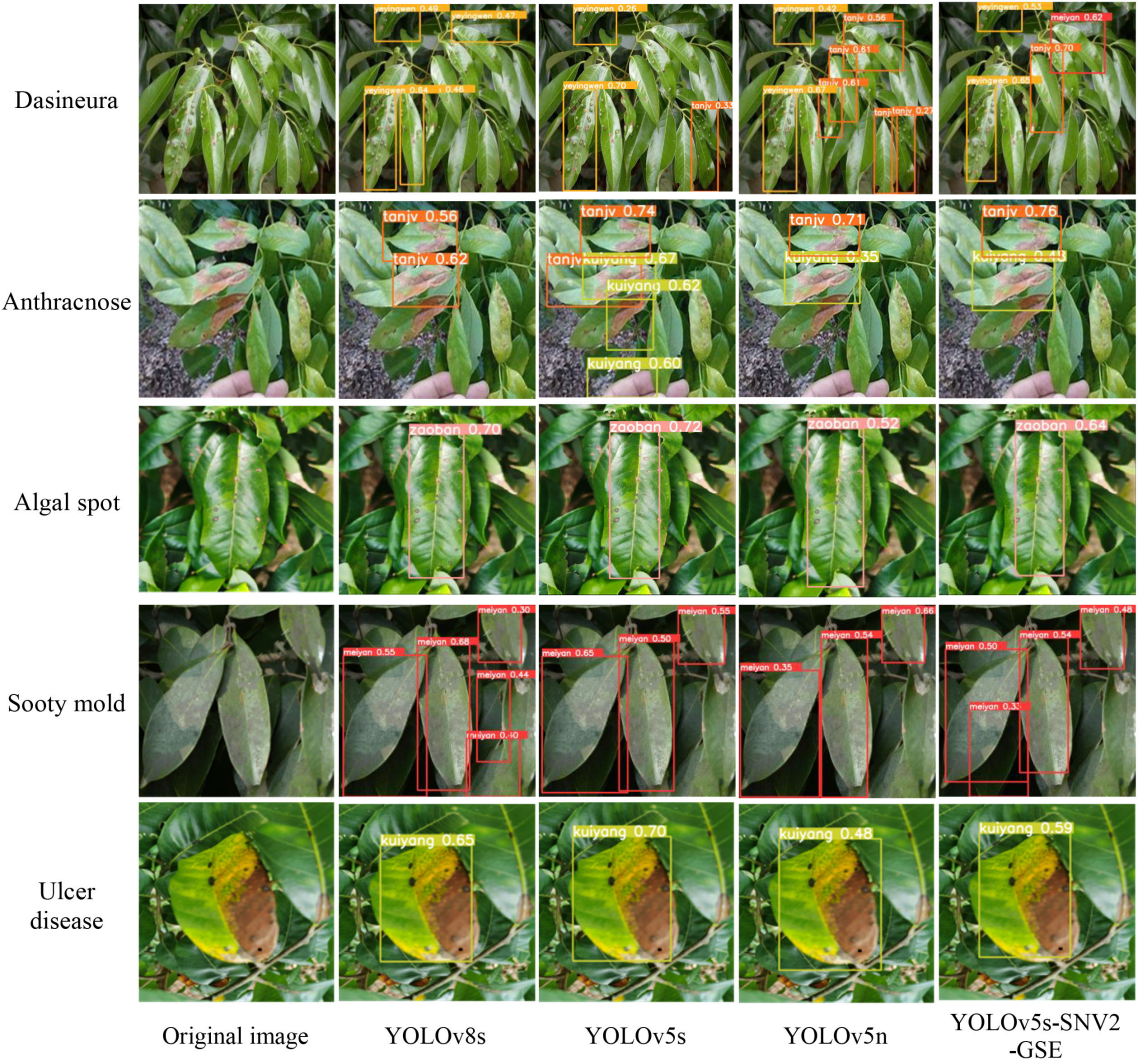
Comparison of model detection results.

Images representative of pests and diseases categories, were selected for evaluation. The three aforementioned models, along with the proposed YOLOv5s-SNV2-GSE, were tested for comparative analysis, as illustrated in **Figure 8**. As could be observed from the results, YOLOv8s achieved relatively higher detection accuracy and confidence scores. However, the confidence of the YOLOv5s-SNV2-GSE model was lower than that of YOLOv8s and YOLOv5s. In the detection of Anthracnose, misdetections occurred in all models except YOLOv8s, as the late-stage Ulcer disease caused leaf wilting, which affected detection. In the detection of Dasineura, the YOLOv5s-SNV2-GSE model suffered from misclassification, where regions with low background brightness were erroneously identified as Sooty mold. Overall, the YOLOv5s-SNV2-GSE model exhibited good confidence in detecting Sooty mold, Algal spot, and Ulcer disease, performing well in recognition tasks.

## 4 Design of Litchi diseases and pests detection system

### 4.1 System construction

The Raspberry Pi main control unit was the core component of the system, running the YOLO algorithm to detect and identify Litchi diseases and pests from the video stream captured by the camera. The Camera Serial Interface (CSI) camera module connected via the Raspberry Pi’s CSI interface, enabling real-time capture of video data from the camera. The system was powered by an independent power source to ensure the portability of the entire setup. For the algorithm, the YOLOv5s-SNV2-GSE model was first trained on an AutoDL server and then transferred to the Raspberry Pi for execution.

### 4.2 System introduction

The embedded main control board of this system used the Raspberry Pi 4B, with the edge computing chip serving as the upper-level control unit. The device was equipped with dual-band WIFI, Bluetooth 5.0, gigabit Ethernet, and USB 3.0 interfaces. It supported 4K dual-screen display output and H.265 hardware video decoding, while maintaining excellent expandability via the 40-pin GPIO interface. Despite its compact size, similar to a credit card, the Raspberry Pi 4B offered performance close to entry-level personal computer (PC) and was widely used in fields such as the Internet of Things, embedded development, and edge computing.

**Figure 9:**
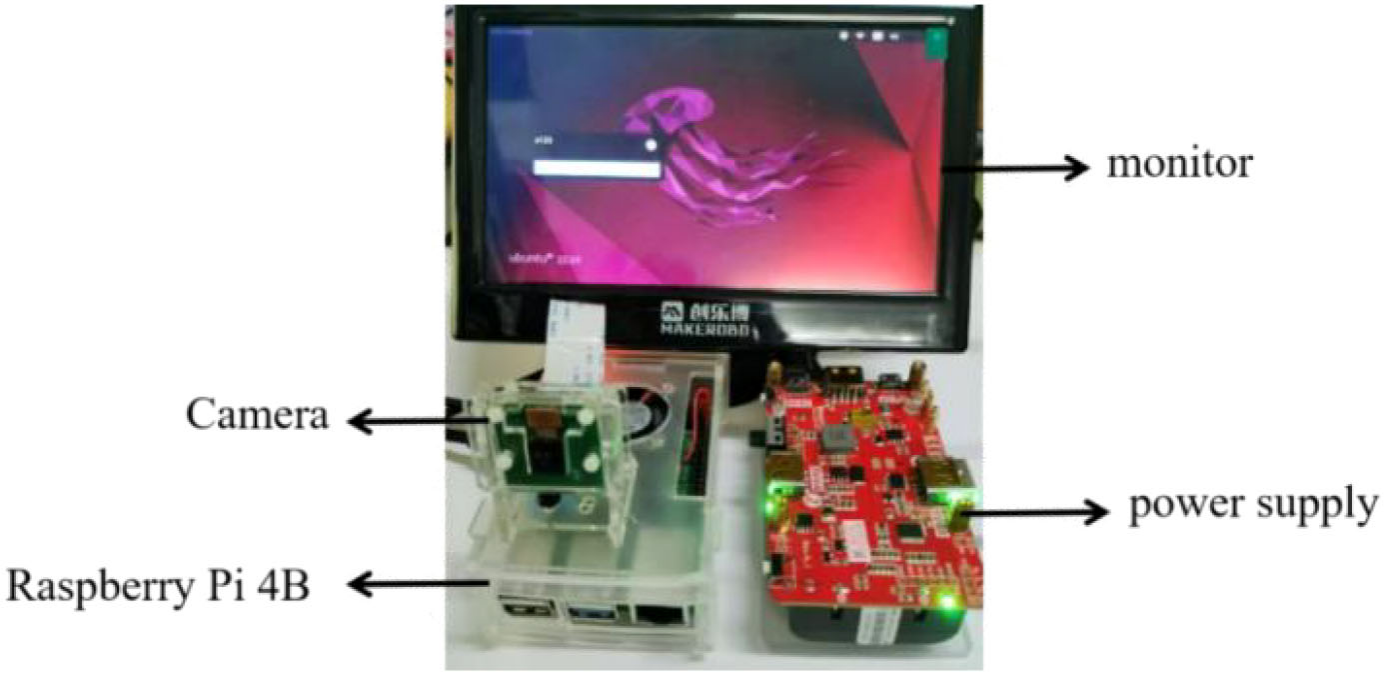
System hardware.

The recognition device used a CSI camera, with the trained model deployed on the Raspberry Pi control board for identification. The recognition data was monitored and analyzed through the backend system. The CSI camera transmitted video streams to the Raspberry Pi, enabling the detection of Litchi diseases and pests. The CSI power supplied powers the Raspberry Pi development board through the charging port, ensuring the system operates normally. The system hardware is shown in **Figure 9**.

### 4.3 System settings

**Figure 10:**
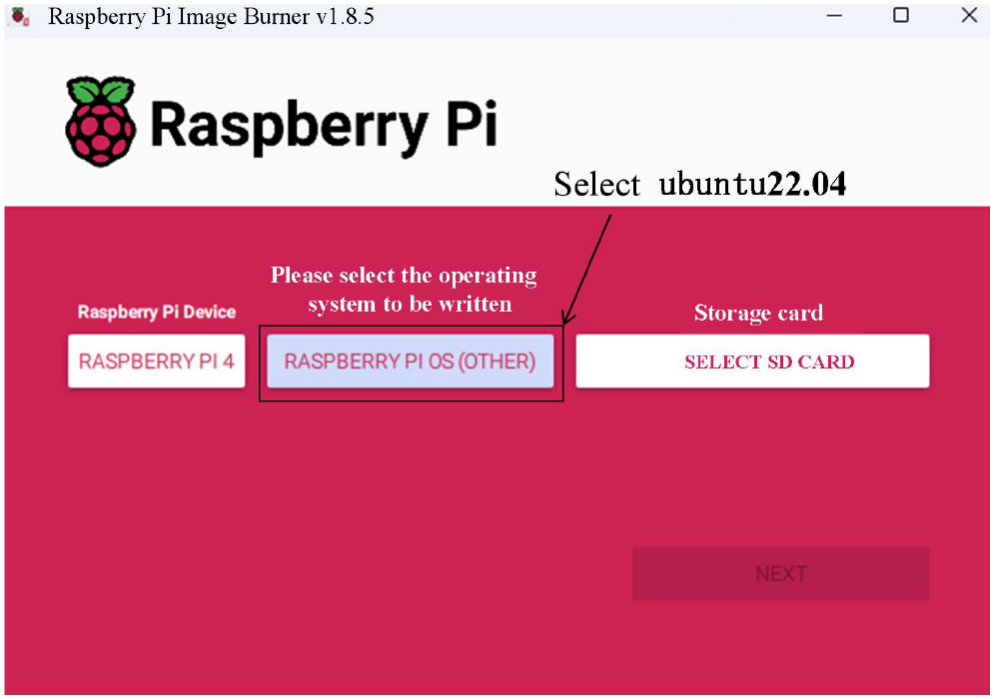
Raspberry Pi system installation.

The improved model in this paper was deployed on the Raspberry Pi 4B. After the hardware setup was completed, the operating system needed to be burned into the hardware. For this, the Ubuntu 22.04 operating system was selected and burned into the Raspberry Pi 4B, as shown in **Figure 10**. Once the system installation was complete, system settings needed to be configured to enable the camera. To did this, opened the terminal command line and entered “sudo raspi-config” to access the system settings interface. Then, selected the “Interface Options” and restarted the Raspberry Pi to enable the camera. This step ensured that the GPU firmware operates properly and that the GPU had allocated sufficient memory space for the camera to function correctly.

### 4.4 Model deployment

Downloaded the YOLOv5s code on the Raspberry Pi system and configured the necessary environment. The “requirements.txt” file specified the required versions of various dependencies. To set up the environment, used the command “pip install -r requirements.txt” in the terminal. Uploaded the trained model file to the Raspberry Pi, adjusted the parameters to set the weight file path, and configured the camera source to 0. Once the camera was enabled, the system would begin capturing footage. Running the program would initiate the YOLOv5 model on the Raspberry Pi.

To evaluate the performance of the deployed models, both the original YOLOv5s and the improved YOLOv5s-SNV2-GSE were tested on the Raspberry Pi hardware. Four video segments, recorded with a mobile phone in a Litchi orchard, were selected for testing. The total video duration was 27 seconds, with a resolution of 3840x2160 pixels. The video was downsampled to 640×640 pixels before being input into the model. The detection results of the video are compared in **Table 6**.

**Table 6:** Model deployment detection results.

Model	FLOPs/G	Parameters/*10^6^	Model size/MB	Frame rate/FPS
YOLOv5s	16	7.03	16	2.1
YOLOv5s-SNV2-GSE	2	0.93	7.1	3.3

**Figure 11:**
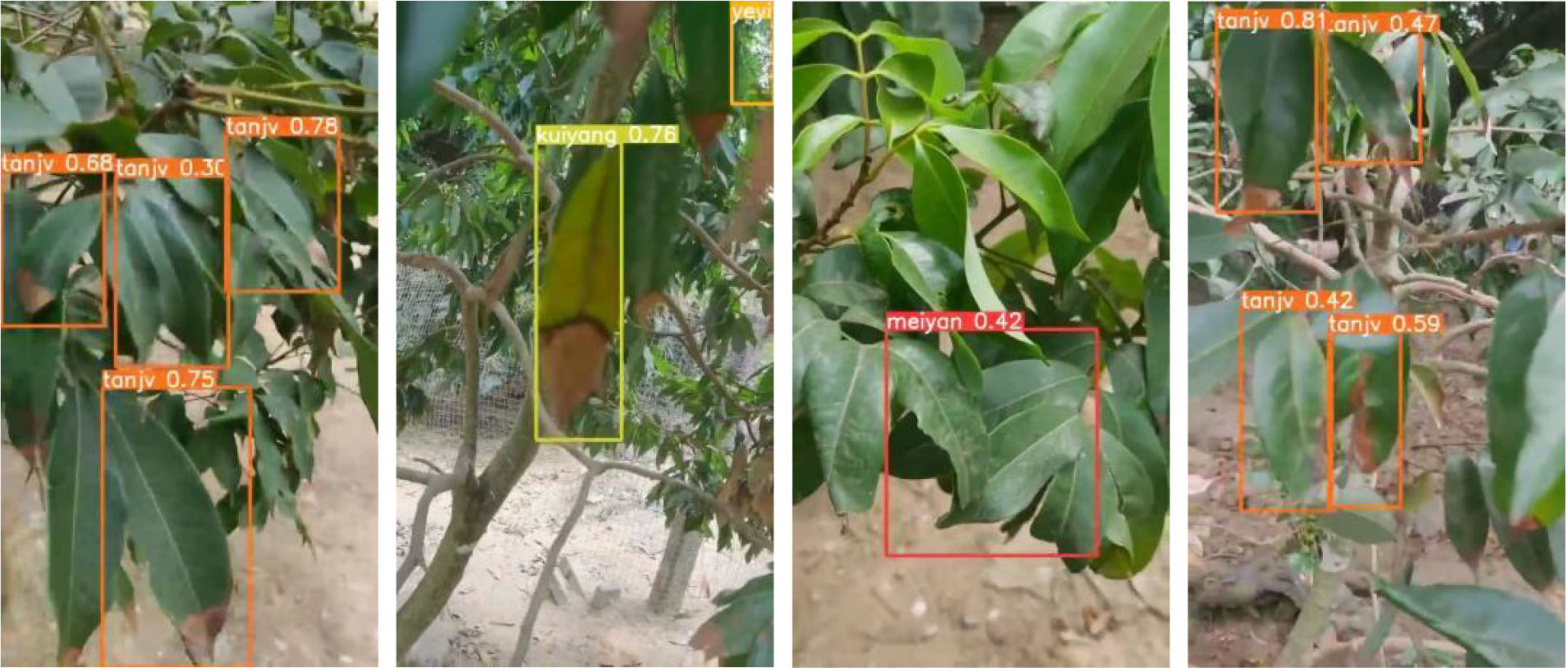
Detection video image.

The improved model offered a significant advantage in detection speed compared to the original model. The frame rate on the Raspberry Pi 4B increased from 2.1 frames per second (FPS) to 3.3 FPS, an improvement of 57.1%. **Figure 11** shows four frames extracted from the detection video. After deploying the model on the Raspberry Pi hardware, real-time detection of Litchi diseases and pests could be achieved. As shown in **Figure 11**, the system effectively identified pests and diseases, and the YOLOv5s-SNV2-GSE model offered low computational cost and memory usage, making it suitable for application on resource-constrained embedded devices.

## 5 Discussion and conclusion

### 5.1 Discussion

This paper proposed an improved YOLOv5s-SNV2-GSE model, which was successfully deployed on embedded platforms to address the issue of real-time detection of Litchi pests and diseases. By incorporating optimization strategies such as ShuffleNetV2, DWConv, and C3Ghost, the model significantly reduced computational complexity and parameter size, enhancing detection accuracy, especially on resource-constrained devices. However, despite the promising results in specific scenarios, there remain many avenues for exploration and further optimization. The dataset was created using standardized Litchi leaf samples under controlled lighting, overlooking real-world factors like weather, dust, and overlapping foliage, which affect accuracy. Additionally, the Raspberry Pi 4B’s passive cooling struggles in hot orchards, causing thermal throttling that reduces system stability and responsiveness.

Future optimization efforts will focus on exploring newer versions of the YOLO series (e.g., YOLOv9, YOLOv10) or other typical two-stage object detection algorithms, with the aim of enhancing both the accuracy and real-time performance of the Litchi pests and diseases detection system (Abulizi et al., 2025; Ye et al., 2024). Furthermore, given the diversity in Litchi cultivation regions and growth environments, optimizing transfer learning strategies and model adaptability is critical to improving the model’s generalization ability and robustness (Fu et al., 2025; Li et al., 2025). Finally, considering the requirements for detection accuracy and feedback efficiency, plans are in place to adopt more powerful edge computing devices (such as Jetson Nano) to replace the Raspberry Pi 4B, which is expected to further enhance the overall performance of the system.

### 5.2 Conclusion

The advancement of modern agriculture necessitates more efficient and scalable solutions for pests and diseases detection. Traditional manual inspection methods are labor-intensive, time-consuming, and often insufficient for real-time diagnosis. To address these limitations, this paper proposed a deep learning-based system for real-time detection of Litchi pests and diseases.

Central to this system was YOLOv5s-SNV2-GSE, a lightweight and efficient enhanced version of YOLOv5s tailored for real-time object detection in agricultural scenarios. Experimental results showed that the YOLOv5s-SNV2-GSE model reduced computational load and parameter count by 88.1% and 87.9%, respectively, compared to the original YOLOv5s. With a compact size of 7.1 MB and a high mAP of 96.7%, it achieved superior performance over lightweight counterparts such as YOLOv5n and YOLOv8s, striking a better balance between accuracy and computational efficiency. Additionally, deployed on a Raspberry Pi 4B, the detection system achieved an inference frame rate of 3.3 FPS, which was 57.1% higher than that of the original YOLOv5s.

For future research, efforts will focus on enhancing the system’s robustness under challenging field conditions, optimizing hardware for improved energy efficiency to support large-scale deployment. these measures are expected to further improve detection accuracy and narrow the performance gap between laboratory tests and real-world agricultural applications.

## Data Availability

The raw data supporting the conclusions of this article will be made available by the authors, without undue reservation.
